# Splicing factor SRSF1 negatively regulates alternative splicing of *MDM2* under damage

**DOI:** 10.1093/nar/gkv223

**Published:** 2015-04-06

**Authors:** Daniel F. Comiskey, Aishwarya G. Jacob, Ravi K. Singh, Aixa S. Tapia-Santos, Dawn S. Chandler

**Affiliations:** 1 Department of Pediatrics, The Ohio State University, Columbus, OH 43210, USA; 2 Center for Childhood Cancer, The Research Institute at Nationwide Children's Hospital, 700 Childrens Drive WA5023, Columbus, OH 43205, USA

## Abstract

Genotoxic stress induces alternative splicing of the oncogene *MDM2* generating *MDM2-ALT1*, an isoform attributed with tumorigenic properties. However, the mechanisms underlying this event remain unclear. Here we explore *MDM2* splicing regulation by utilizing a novel minigene that mimics endogenous *MDM2* splicing in response to UV and cisplatinum-induced DNA damage. We report that exon 11 is necessary and sufficient for the damage-specific alternative splicing of the *MDM2* minigene and that the splicing factor SRSF1 binds exon 11 at evolutionarily conserved sites. Interestingly, mutations disrupting this interaction proved sufficient to abolish the stress-induced alternative splicing of the *MDM2* minigene. Furthermore, SRSF1 overexpression promoted exclusion of exon 11, while its siRNA-mediated knockdown prevented the stress-induced alternative splicing of endogenous *MDM2*. Additionally, we observed elevated SRSF1 levels under stress and in tumors correlating with the expression of *MDM2-ALT1*. Notably, we demonstrate that *MDM2-ALT1* splicing can be blocked by targeting SRSF1 sites on exon 11 using antisense oligonucleotides. These results present conclusive evidence supporting a negative role for SRSF1 in *MDM2* alternative splicing. Importantly, we define for the first time, a clear-cut mechanism for the regulation of damage-induced *MDM2* splicing and present potential strategies for manipulating MDM2 expression via splicing modulation.

## INTRODUCTION

Alternative splicing is an important cellular process that contributes to proteome diversity. It is estimated that >95% of all genes undergo alternative splicing (1–4). These alternative splicing events are often spatially and temporally regulated and generated in response to external stimuli (5–13). In general, the regulation of alternative splicing is achieved through complex interplay between *cis* regulatory elements within the pre-mRNA and the *trans* protein factors that bind them. *Trans*-binding protein factors belong to two general classes: serine-arginine rich (SR) proteins and heterogenous ribonucleoproteins (hnRNPs), whose canonical roles are to either promote or repress the inclusion of an exon in the nascent pre-mRNA transcript, respectively (14–16). The balance in the levels of these factors and their binding to specific sites on the pre-mRNA are key toward influencing the decisions of the spliceosome, thereby enabling splicing regulation. SRSF1, formerly SF2/ASF, is one such member of the serine-arginine rich family of SR proteins. In addition to its role in alternative splicing, SRSF1 is required to mediate canonical splicing events including 5′ splice site selection and lariat formation of the major spliceosome (17,18). SRSF1 is an important proto-oncogene due to its role in the alternative splicing regulation of several cancer-associated genes (19). Here we describe a role for SRSF1 in the regulation of *MDM2* splicing.

Murine Double Minute 2 (MDM2) is an E3 ubiquitin ligase and negative regulator of the tumor suppressor protein p53. Under normal conditions, *MDM2* is constitutively spliced to generate a full-length protein, which self-dimerizes and promotes the proteasome-mediated degradation of p53 (20–25). However, under stress *MDM2* undergoes alternative splicing, generating splice variants that are unable to bind and regulate p53 (10,26,27). Subsequently, p53 becomes upregulated and activates downstream targets involved in apoptosis and cell cycle arrest (28–30). *MDM2-ALT1*, which consists of only the two terminal coding exons 3 and 12, is the most frequently observed of these splice isoforms. Despite studies characterizing *MDM2-ALT1* as a dominant negative regulator of full-length *MDM2* and its pervasiveness in various cancers (31–36), there is very little known about the regulation of *MDM2* alternative splicing in cancer and under stress.

What we currently know is that *MDM2* splicing occurs in cells in response to UV irradiation and cisplatinum treatment in a manner independent of the p53, ataxia telangiectasia mutated (ATM), and ataxia telangiectasia and Rad3-related protein (ATR) status of these cells (10). Additionally, cotranscriptional regulation of *MDM2* splicing has been demonstrated in response to camptothecin. In this case, the disruption of the interaction between the Ewing's Sarcoma Protein (EWS) with RNA Polymerase II (Pol II) and the spliceosome-associated factor Y-box-binding Protein 1 (YB-1) upon camptothecin treatment results in the uncoupling of transcription and splicing, and ultimately the alternative splicing of *MDM2* (25). However, *MDM2* alternative splicing can also occur independently of transcription as demonstrated by *in vitro* cell-free splicing systems that utilize nuclear extracts from normal, UV, or cisplatinum-treated cells (37). Using such *in vitro* splicing assays in conjunction with a stress-responsive *MDM2* minigene, we previously identified conserved positive sequences within intron 11 of *MDM2* and binding factors such as FUBP1 that are important for its efficient splicing (37,38).

In the present study we report for the first time repressive elements in *MDM2* exon 11 that facilitate its damage-inducible alternative splicing. Using a SELEX-based bioinformatics program, we identified predicted binding sites for SRSF1 in this regulated exon. We report that the binding of SRSF1 to this site is increased under damage and its mutation is sufficient to ablate damage-induced exon 11 exclusion in a three-exon minigene system in cell-based transfection assays. Additionally we show that blocking this binding site on endogenous *MDM2* is capable of preventing the generation of *MDM2-ALT1* under stress. Altogether our data address SRSF1 as a critical modulator of endogenous *MDM2* alternative splicing, providing necessary information in the regulation of this important oncogene and a potential therapeutic target for intervention in the myriad cancers in which *MDM2-ALT1* is observed.

## MATERIALS AND METHODS

### Plasmids, protein expression constructs

*LacZ* cDNA was cloned into the BglII-XhoI sites of the Cre-inducible pCCALL2 vector whose β-galactosidase and neomycin resistance cassettes were previously excised by Cre recombinase to facilitate constitutive expression of the corresponding downstream cDNA. *HNRNPL* cDNA was cloned into the pcDNA3 vector. The p3x-FLAG hnRNPF and pFRT/TO/HIS/FLAG/HA-hnRNPR plasmids were purchased commercially from Addgene. The FLAG-GFP-hnRNPU construct was provided as a kind gift from Dr. Patrick Calsou. The FLAG-hnRNPD construct was provided as a kind gift from Dr. Stephen Kolb. The T7-SRSF1 construct was provided as a kind gift from Dr. Adrian Krainer.

### Minigene constructs

The *MDM2* 3-11-12*s* minigene was constructed by truncating exon 3 (from 85 nt to include only the 38 nt at its 3′ end), exon 12 (from 229 nt to include only the 73 nt at the 5′ end), the upstream intron 3/10 (from 167 nt to 72 nt retaining 19 nt at its 5′ end and 53 nt of the 3′ end), and the downstream intron 11 (from 316 nt to 147 nt including only 79 nt at the 5′ end and 68 nt of the 3′ end) of the previously described *MDM2* 3-11-12 stress-responsive minigene (37). To assemble this minigene into the pCMV-tag2B vector, a strategy similar to the one described for the construction of the 3-11-12 minigene (37) was adopted. Using restriction sites engineered into the 5′ ends of polymerase chain reaction (PCR) products, the 3′ end of intron 11 (68 nt region) and exon 12 (the complete exon 12 from the 3-11-12 minigene) were first cloned into the EcoR1-Xho1 sites of the pCMV-Tag2B vector using the following primers: For: 5′ TCGAATTCGCTAGCATTCCTGTGACTGAGCAG 3′ and Rev: 5′ TAACTCGAGCCTCAACACATGACTCT 3′. Following this, exon 12 was truncated at its 3′ end first by restriction digest of the ApaI site in the multiple cloning site (MCS) of the pCMV-tag2B vector and the ApaI site native to exon 12 to release the 3′ fragment of exon 12. Following this, the construct was relegated to obtain the short exon 12 with only 73 nt at the 5′ end. Subsequently, the 3′ end of intron 3/10 (53 nt), exon 11 (78 nt) and the 5′ end of intron 11 (79 nt) were amplified using primers (For: 5′ GCCTGCAGCTGATTGAAGGAAATAGGGCG and Rev: 5′ AGGGAATTCGAAGCTAGATATAGTCT 3′) that bear PstI and EcoRI sites at their 5′ ends and the PCR product obtained was cloned into the PstI–EcoRI sites of construct bearing the other end of intron 11 and truncated exon 12. Finally, using a similar approach, the exon 3 (38 nt) and the 5′ end of intron 3/10 (19 nt) were amplified (For: 5′ GCGGATCCCCACCTCACAGATTCCAGCTTCGG 3′ Rev: 5′ CTGCAGCAAAAATACTAACCAGGGTCTC 3′) and cloned into the BamHI and PstI sites located on the MCS of the assembly vector containing the rest of the minigene. The construction of the *p53* 7-8-9 minigene has been described previously (37).

#### Chimeric minigenes

The chimeric minigenes of *MDM2* or *p53* origin were all constructed by keeping the terminal exons (3 and 12 for the *MDM2* and 7 and 9 for the *p53* minigenes) intact with respect to their wild-type (WT) counterparts. Also, when the intronic regions were swapped between the *MDM2* and *p53* minigenes, they did not include their native splice sites (the first 10 and the last 10 nt of each intron were considered as the splice sites and were not included in the intronic region ligated into the heterologous system). On the other hand, the splice sites were maintained native to the exons (native to either the terminal exons or the internal exon being swapped) as 10 nt in the intron upstream or downstream or flanking the exon. For instance, exon 11 retained the splice sites native to *MDM2* with the flanking 10 nt from intron 11 and intron 3/10 even when placed in the context of the *p53* minigene. A similar condition was maintained when *p53* exon 8 was placed in the *MDM2* minigene context. The chimeric minigenes were assembled in the BamHI and HindIII sites of the pCMV-tag2B vector using the Clontech Infusion HD Kit (Catalog Number 638909). The individual elements to be assembled were first amplified using primers (designed using the Infusion HD primer-design tools) with 15 bp overhangs complementary to the elements placed adjacent to them. Following this, the inserts were ligated into the pCMV-Tag2B vector, digested with BamHI and HindIII, and then transformed into stellar competent cells according the manufacturer's protocols. All clones were verified by DNA sequencing.

### Protein extraction from RMS tissues

Human tissue samples were obtained from the Cooperative Human Tissue Network, Pediatric Division at Nationwide Children's Hospital after Institutional Review Board approval. All specimens were snap-frozen and stored at −80°C. The tissue was ground using a mortar and pestle in liquid nitrogen. Protein was extracted using 300 μl of RIPA buffer (150 mM NaCl, 50 mM Tris pH 8.0, 0.5% sodium deoxycholate, 1.0% Triton X-100, 0.1% sodium dodecyl sulfate [SDS], 1 mM ethylenediaminetetraacetic acid pH 8.0) and homogenized with a Tissumizer (Tekmar, Cincinnati, OH, USA).

### RT and PCRs

Typical reverse transcription (RT) reactions were carried out using 1 μg of RNA unless otherwise mentioned. Transcriptor RT enzyme (Catalog No. 03531287001) from Roche Diagnostics (Indianapolis, IN, USA) was used for the cDNA synthesis reactions according to the manufacturer's instructions. PCRs for *in vitro* splicing were performed using Platinum *Taq* Polymerase (Catalog Number 11304-011) from Life Technologies (Carlsbad, CA, USA) and subjected to a 25-cycle PCR using ATP γ-^32^P-radioactively-labeled Flag primer and gene-specific reverse primers under standard PCR conditions (95°C 5′, 95°C 0:40, 55°C 0:30, 72°C 1′, 72°C 7′). Endogenous *MDM2* PCRs were performed using *Taq* Polymerase (Catalog Number D6677) from Sigma-Aldrich (St. Louis, MO, USA) using a set of nested primers as previously reported (39). *SRSF1* isoform PCRs were performed using Platinum *Taq* Polymerase (Catalog Number 11304-011) from Life Technologies (Carlsbad, CA, USA) and subjected to a 30-cycle PCR using primers (SF2-e3F 5′ CACTGGTGTCGTGGAGTTTGTACGG 3′ and SF2-e4R 5′ GGGCAGGAATCCACTCCTATG 3′) under standard PCR conditions (94°C 5′, 94°C 0:30, 62°C 0:30, 72°C 2′, 72°C 7′). *SRSF1* and *CDKN1A* qPCRs were performed using TaqMan^®^ Universal PCR Master Mix (Catalog Number 4304437) from Life Technologies (Carlsbad, CA, USA) using probes for *SRSF1* (Hs001199471), *CDKN1A* (Hs00355782), and *GAPDH* (Hs503929097) under standard PCR conditions (95°C 15′, 95°C 0:15, 60°C 1′) for 40 cycles on an Applied Biosystems 7900HT Fast Real Time PCR system (Life Technologies, Carlsbad, CA, USA).

### Western blot analysis and antibodies

Cell were lysed in NP-40 buffer and equal amounts of protein were loaded in 6× SDS sample buffer onto a sodium dodecyl sulfate-polyacrylamide gel (SDS-PAGE), blotted onto a polyvinylidene difluoride (PVDF) membrane, and analyzed for expression of SRSF1 (Catolog Number 32-46000) from Novex by Life Technologies (Carlsbad, CA, USA) or T7-Tag (Catalog Number 69522) from EMD Millipore (Merck KGaA, Darmstadt, Germany). For detection of LacZ, MYC-tag clone 9E10 (Catalog Number sc-40) from Santa Cruz Biotechnology (Dallas, TX, USA) was used. For detection of p3x-FLAG-HNRNPD and FLAG-hnRNPD, ANTI-FLAG clone M2 (Catalog Number F1804) from Sigma-Aldrich (St. Louis, MO, USA) was used. To detect expression of pFRT/TO/HIS/FLAG/HA-hnRNPR, anti-HA High Affinity (Catalog Number 11867423001) from Roche Diagnostics (Indianapolis, IN, USA) was used. For detection of FLAG-GFP-hnRNPU, anti-GFP (Catalog Number ab13970) from Abcam (Cambridge, MA, USA) was used. To detect expression of β-Actin clone AC-15 (Catalog Number A5441) from Sigma (St. Louis, MO, USA) was used. For detection of β-tubulin expression, clone E7 was used from a hybridoma. Protein sizes were determined using the Precision Plus Protein Dual Color Standards marker (Catalog Number 161-0374) from Life Technologies (Carlsbard, CA, USA).

### RNA oligonucleotide pull down

RNA probes were synthesized from Integrated DNA Technologies (Coralville, IA, USA) (SRSF1-WT ‘UAUCAGGCAGGGGAGAGUGAU’ and SRSF1-MUT ‘UAUCAGAAAGGGGAGAGUGAU’). A total of 5 nmol of RNA was modified and purified in a 400 μl reaction containing 100 mM NaCH_3_COO^−^, 5 mM NaIO_4_, pH 5.0 for 1 h in the dark. RNA was ethanol precipitated and resuspended in 50 μl 0.1 M NaCH_3_COO^−^, pH 5.0. Adipic acid dihydrazide agarose beads (Catalog Number A0802-10ML) from Sigma (St. Louis, MO, USA) were washed 4× in 0.1 M NaCH_3_COO^−^ and incubated with RNA overnight at 4°C on rotator. Bead-conjugated was washed successively 3× in 2 M NaCl, then Buffer D (20 mM HEPES-KOH pH 8.0, 20% glycerol, 0.1 M KCl, 0.2mM ethylenediaminetetraacetic acid, 0.5 mM dithiothreitol [DTT]) spinning 300 rpm and resuspended in 62.5 μl Buffer D. RNA was then incubated in a splicing reaction at 30°C for 40 min, gently mixing every 5 min. Protein-bound beads were washed 3× in Buffer D, then eluted in 40 μl 2× SDS Buffer. Beads were boiled 100°C for 5 min, then spun down 10 000 rpm at 4°C for 10 min. Eluates were collected and loaded in equal volume on 10% SDS-PAGE gel, transferred to PVDF membrane, and probed for SRSF1 (1:1000) and β-Actin (1:250 000).

### *In vitro* splicing

Pre-mRNA was transcribed *in vitro* with T7 MEGAscript (Catalog Number AM1334) by Ambion by Life Technologies (Carlsbad, CA, USA) using PCR templates amplified from the various *MDM2* or *p53* minigenes incorporating a T7 promoter region and a Flag-tag region at the 5′ end. The primers utilized were to amplify PCR products for use as templates for the *in vitro* transcription were as follows: for the *MDM2* 3-11-12*s* and the *MDM2*-based chimeric minigenes: For: 5′ AGTAATACGACTCACTATAGGGATTACAAGGATGACGACGATAAGAGCCCGGGCGGATCCCCACCTCACAGATTC 3′ and Rev: 5′ ACTTACGGCCCAACATCTGTTGCAATGTGATGG 3′ with a 5′ splice site and the primers for the *p53* 7-8-9 minigene and the *p53*-based chimeric minigenes were as follows: For: 5′ AGTAATACGACTCACTATAGGGATTACAAGGATGACGACGATAAGGTTGGCTCTGACTGTACCACCATC 3′ and Rev: 5′ ACTTACGGCTGAAGGGTGAAATATTCTCCATCC 3′ with a 5' splice site at the end. A total of 20 fmol of the *MDM2* and *p53* minigene *in vitro-*transcribed RNA was subjected to *in vitro* splicing at 30°C in nuclear extracts from normal or 12 h cisplatinum-damaged HeLa S3 cells as previously described (37). RNA was extracted by standard phenol/choloroform and precipitated with 100% ethanol. RNA was reverse transcribed and subjected to a 25-cycle PCR as indicated above. PCR products were loaded on a 6% denaturing Urea-PAGE gel, dried at 80°C for 45 min, and exposed to a phosphor screen overnight. The marker used was the radioactively-labeled *in vitro*-transcribed RNA Century Marker (Catalog Number AM7140) from Life Technologies (Carlsbad, CA, USA). The sequences for the gene-specific primers (for the *MDM2* and *p53* minigenes and their corresponding chimeric minigenes) and the Flag-tag primer used have been described (37).

### Ribo-oligonucleotide competition

Splicing reactions with nuclear extracts from 12 h cisplatinum-damaged HeLa S3 cells were pre-incubated at 30°C in the presence of absence of 200 pmol oligonucleotides (SRSF1-WT ‘UAUCAGGCAGGGGAGAGUGAU’ or SRSF1-MUT ‘UAUCAGAAAGGGGAGAGUGAU’) for 1 h. At 1 h 20 fmol of the *MDM2* 3-11-12*s* minigene was added to each reaction and spliced as previously described for 2 h (37).

### Quantification of splicing ratios

**Figure 1.**
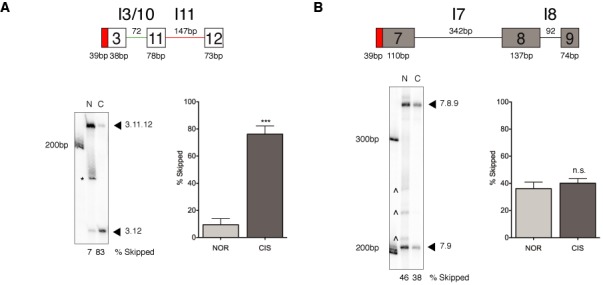
The *MDM2* 3-11-12*s* minigene undergoes damage-induced exon 11 skipping in an *in vitro* splicing system while a control *p53* 7-8-9 minigene remains unresponsive. (**A**) A minimal *MDM2* 3-11-12*s* minigene, constructed to assess the elements essential for the generation of *MDM2-ALT1* alternative splicing, was derived from the previously described *MDM2* 3-11-12 minigene, which is responsive to stress-induced alternative splicing. The schematic represents the 3-11-12*s* minigene and the sizes depicted reflect the length of the exonic and intronic regions of the minigene construct and are inclusive of the Flag-tag and the intervening region (cloning sites) of the pCMV-tag2B vector at the 5′ end of the minigene construct. *In vitro*-transcribed RNA obtained from the minigenes was subjected to a cell-free *in vitro* splicing assay using nuclear extracts from either normal (N, NOR) or cisplatinum-treated HeLa S3 cells (C, CIS). RNA was isolated, reversed transcribed and subjected to a 25-cycle PCR using γ-^32^P-radioactively-labeled Flag primer and gene-specific reverse primers. The *MDM2* minigene predominantly skips internal exon 11 when spliced in nuclear extracts from cisplatinum-treated cells, but not in nuclear extract from normal cells. The bar graphs represent the percentage of 3.12 skipped product obtained from three independent *in vitro* splicing experiments under each condition and the error bars represent standard error mean (SEM). The difference in the percentage of 3.12 product between normal and damaged splicing conditions is statistically significant (*n* = 3). *Indicates non-specific band also seen in –ATP controls (see Supplementary Figure S1). ^∧^Indicates probable PCR degradation products. (**B**) Damage-responsive alternative splicing is transcript-specific. A *p53* 7-8-9 minigene shows no changes in splicing patterns between the normal and damaged nuclear extract (*n* = 3). The sizes of the minigene depicted in the schematic are reflective of the Flag-tag and vector-specific regions at the 5′ end of the minigene construct in a manner similar to the 3-11-12*s* minigene.

**Figure 2.**
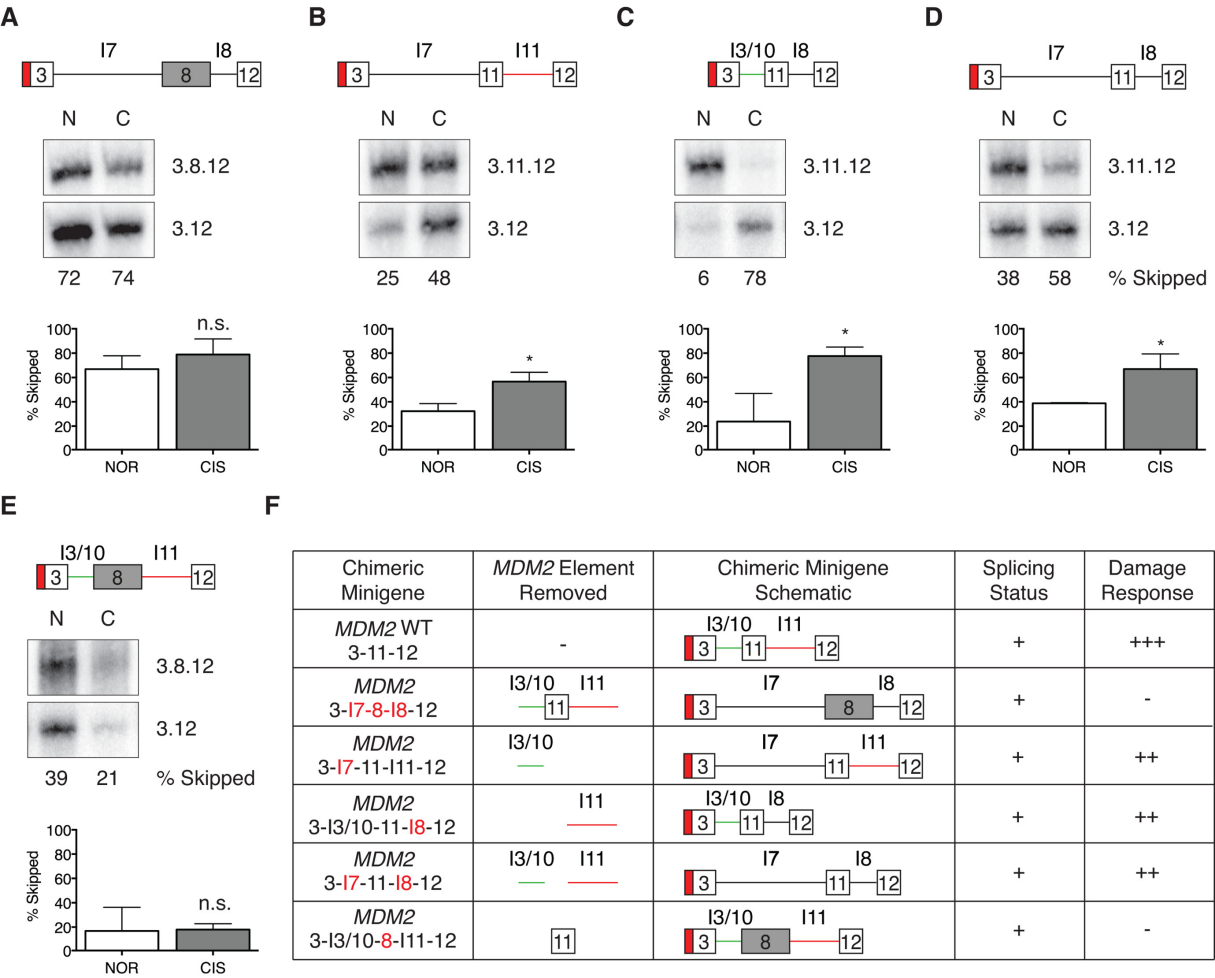
Loss of *MDM2* exon 11 abolishes stress-responsive alternative splicing of the *MDM2* minigene. Chimeric *MDM2* minigenes were created by replacing the introns and/or internal exon of *MDM2* with corresponding regions from the non-stress-responsive *p53* minigene as depicted in the schematics and were subjected to *in vitro* splicing in nuclear extracts from normal (N) or cisplatinum-(C)-treated cells. Percentage of the skipped splicing product 3.12 for the various chimeric minigenes is represented graphically for three independent experiments with error bars representing the SEM. (**A**) The internal exon 11 and the introns of the *MDM2* 3-11-12*s* minigene were removed and replaced with exon 8 and the introns from the *p53* minigene. The damage-responsive alternative splicing of the *MDM2* minigene is abolished and there is no significant difference between the percentage of 3.12 skipped product between normal and damaged conditions (*n* = 3). However, statistically significant changes in the skipping of internal exon 11 (percent 3.12) between the normal and cisplatinum-damaged conditions was observed with the chimeric minigenes. (**B**) The upstream intron of *MDM2* 3-11-12*s* minigene was replaced by the *p53* intron 7 (*n* = 3). (**C**) The downstream intron of *MDM2* 3-11-12*s* minigene was replaced by *p53* intron 8 (*n* = 3). (**D**) Both the introns of *MDM2* 3-11-12*s* minigene were replaced with *p53*'s introns 7 and 8 (*n* = 3) in a manner similar to the WT *MDM2* 3-11-12*s* minigene. (**E**) The chimeric *MDM2* minigene in which exon 11 was removed and replaced with *p53* exon 8 displayed a loss of the damage-responsive alternative splicing and no statistically significant changes were observed in the percentage of 3.12 product obtained under normal and damaged splicing conditions (*n* = 3). (**F**) Table summarizing the *MDM2* minigene constructs and the status of their damage-responsive splicing.

**Figure 3.**
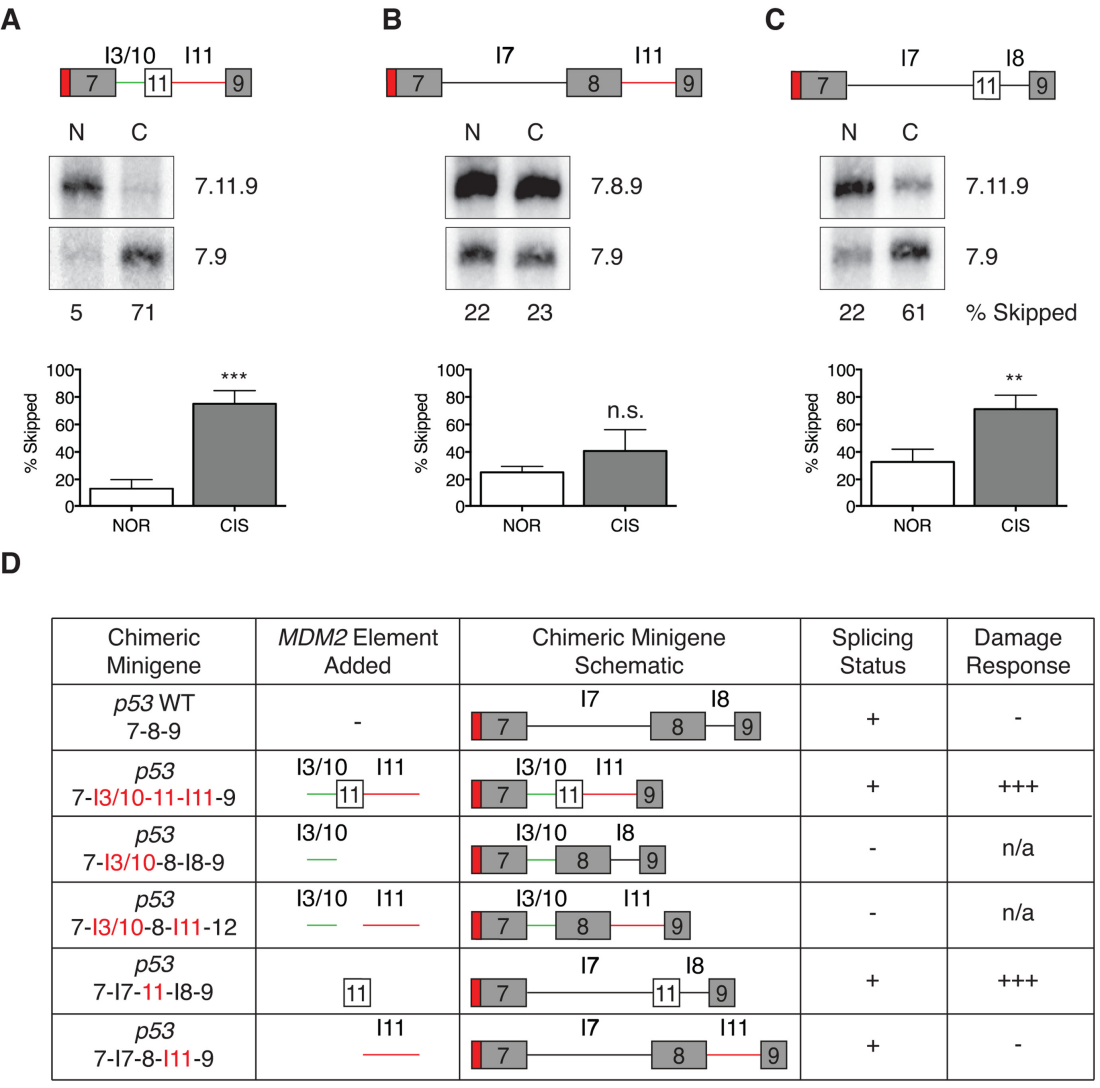
*MDM2* exon 11 is sufficient to regulate stress-responsive splicing in the heterologous *p53* minigene context. Chimeric *p53* minigenes were created by replacing the introns and/or internal exon of *p53* with corresponding regions from the stress-responsive *MDM2* minigene. These minigenes were then spliced *in vitro* in nuclear extracts prepared from normal (N) and cisplatinum-(C)-treated cells. Percentage of the skipped splicing product 7.9 for the various chimeric minigenes is represented graphically for three independent experiments and the error bars reflect the SEM. (**A**) The chimeric construct in which the *p53* minigene's internal exon 8 and its flanking introns were replaced by the corresponding regions of the *MDM2* 3-11-*12s* minigene exhibited damage-specific skipping of the internal exon in a manner similar to the WT *MDM2* 3-11-12*s* minigene. The difference in the percentage of the 7.9 skipped product generated between normal and cisplatinum-damaged conditions was statistically significant (*n* = 3). (**B**) The chimeric *p53* minigene, in which its downstream intron 8 was replaced by intron 11 of the *MDM2* 3-11-12*s* minigene, did not show statistically significant changes in the percentage of 7.9 product obtained as a result of splicing under normal and cisplatinum-damaged conditions (*n* = 3). (**C**) The *in vitro* splicing of the chimeric *p53* minigene containing the exon 11 of *MDM2* minigene in nuclear extracts from normal and cisplatinum-treated cells showed statistically significant damage-specific induction of the 7.9 skipped product in a manner similar to the WT *MDM2* 3-11-12*s* minigene (*n* = 3). (**D**) Table summarizing the *p53* minigene constructs and the status of their damage-responsive splicing. Splicing status of (−) indicates that constructs were not splicing competent in nuclear extracts as fully-spliced products were not detected by RT-PCR.

**Figure 4.**
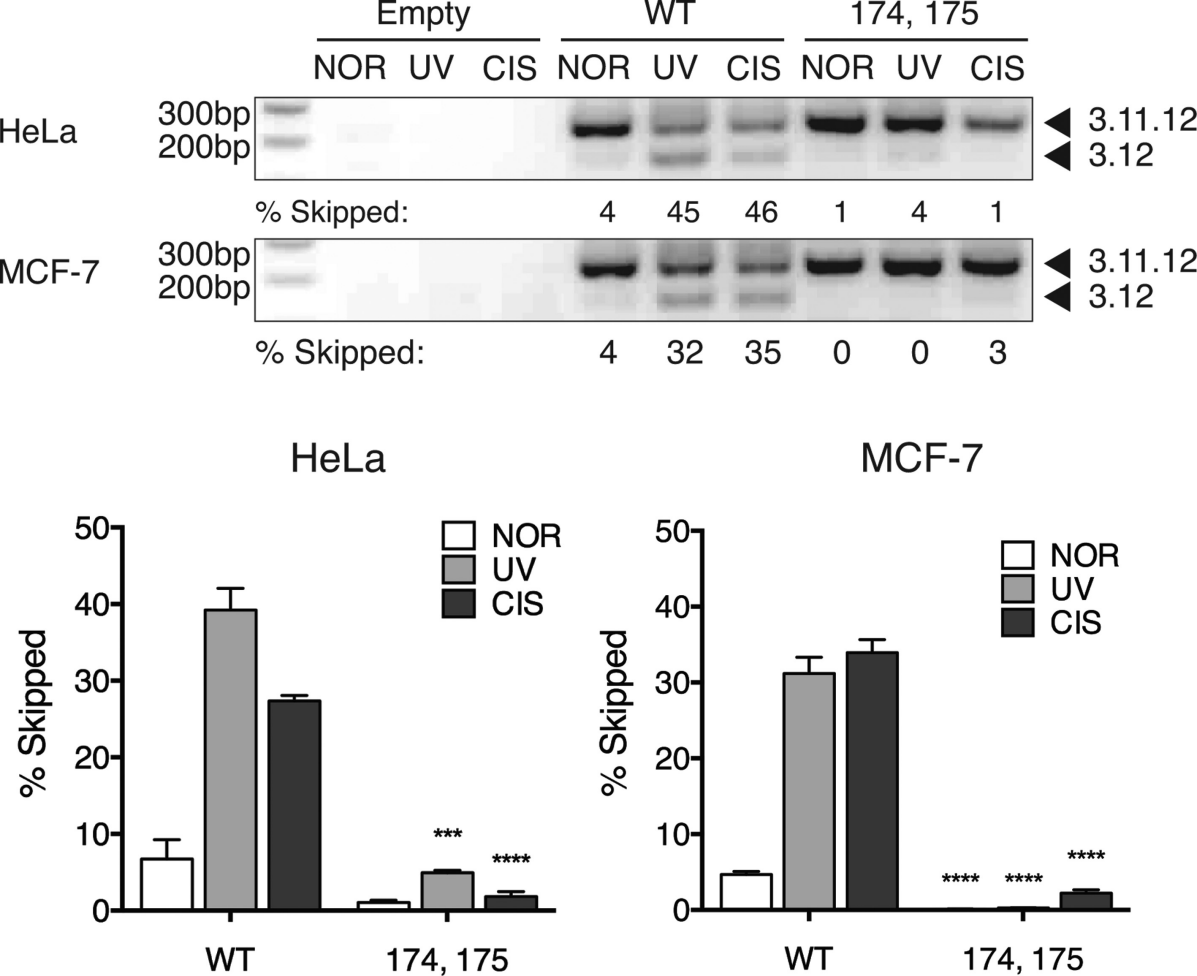
SRSF1 acts a negative regulator of splicing in *MDM2* exon 11. *MDM2* minigenes were transfected into MCF-7 and HeLa cells for 24 h and then treated under normal, 50 J/m^2^ ultra-violet (UVC) or 75 μM cisplatinum(CIS)-damaged conditions for an additional 24 h. RNA was extracted and subjected to RT-PCR using a minigene- and gene-specific primer. PCR products were separated on a 1.5% agarose gel and spliced products were visualized by UV imaging. The bar graphs represent the percentage of 3.12 skipped product obtained from at least three independent experiments under each condition and the error bars represent SEM. The SRSF1 mutant minigene loses damage-induced alternative splicing (MCF-7 *n* = 4, HeLa *n* = 3).

**Figure 5.**
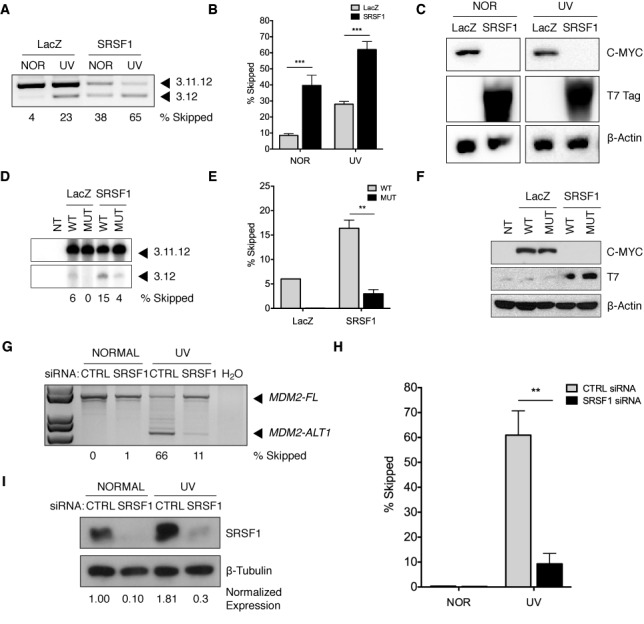
SRSF1 induces exclusion of *MDM2* exon 11. (**A**) LacZ or T7-SRSF1 were cotransfected with the *MDM2* 3-11-12*s* minigene in MCF-7 cells for 24 h and then treated under normal or 50 J/m^2^ ultra-violet (UVC) conditions for an additional 24 h. RNA was extracted and subjected to a RT-PCR using a minigene- and gene-specific primer. PCR products were separated on a 1.5% agarose gel and spliced products were visualized by UV imaging (*n* = 3). (**B**) The bar graphs represent the percentage of 3.12 skipped product obtained from three independent experiments under each condition and the error bars represent SEM. Overexpression of T7-SRSF1 in transfected MCF-7 cells under both normal and UV conditions induced skipping of exon 11 in the WT *MDM2* 3-11-12s minigene compared to the negative control (LacZ). (**C**) Protein lysates were run on a 10% SDS-PAGE gel and probed with C-MYC, T7 and β-Actin antibodies to confirm protein overexpression. (**D**) The *MDM2* minigenes (WT or MUT) and LacZ or T7-SRSF1 were cotransfected in MCF-7 cells for 24 h. RNA was extracted and subjected to a radioactive RT-PCR using a minigene- and gene-specific primer. PCR products were separated on a 6% Urea-PAGE gel and spliced products were visualized by autoradiography (*n* = 3). (**E**) Overexpression of T7-SRSF1 in transfected MCF-7 cells under normal conditions induced skipping of exon 11 in the WT minigene compared to the negative control (LacZ), whereas the SRSF1 mutant was unresponsive to damage induction. Representative data of triplicate experiments is shown. (**F**) Protein lysates were run on a 10% SDS-PAGE gel and probed with C-MYC, T7 and β-Actin antibodies to confirm protein overexpression. (**G**) MCF-7 cells were transfected with either 30 nM of non-specific (CTRL) or SRSF1-specific (SRSF1). At 42 h, cells were split 1:2 and at 48 h were cultured either normally or treated with 50 J/m^2^ ultra-violet (UVC) for 24 h. At 72 h post-transfection, cells were harvested for RNA and protein. RNA was reverse transcribed and subjected to a nested PCR. PCR products were separated on a 1.5% agarose gel and spliced products were visualized by UV imaging. The percent of *MDM2-ALT1* is shown relative the amount of full-length *MDM2* (*MDM2-FL*). The bar graph (**H**) represents the percentage of *MDM2-ALT1* skipped product obtained from three independent experiments and the error bars represent SEM. Upon knockdown of SRSF1, endogenous *MDM2* loses damage-inducible expression of *MDM2-ALT1* (*n* = 3). (**I**) Protein lysates were run on a 10% SDS-PAGE gel and probed with SRSF1 and β-Tubulin antibodies to confirm protein knockdown.

**Figure 6.**
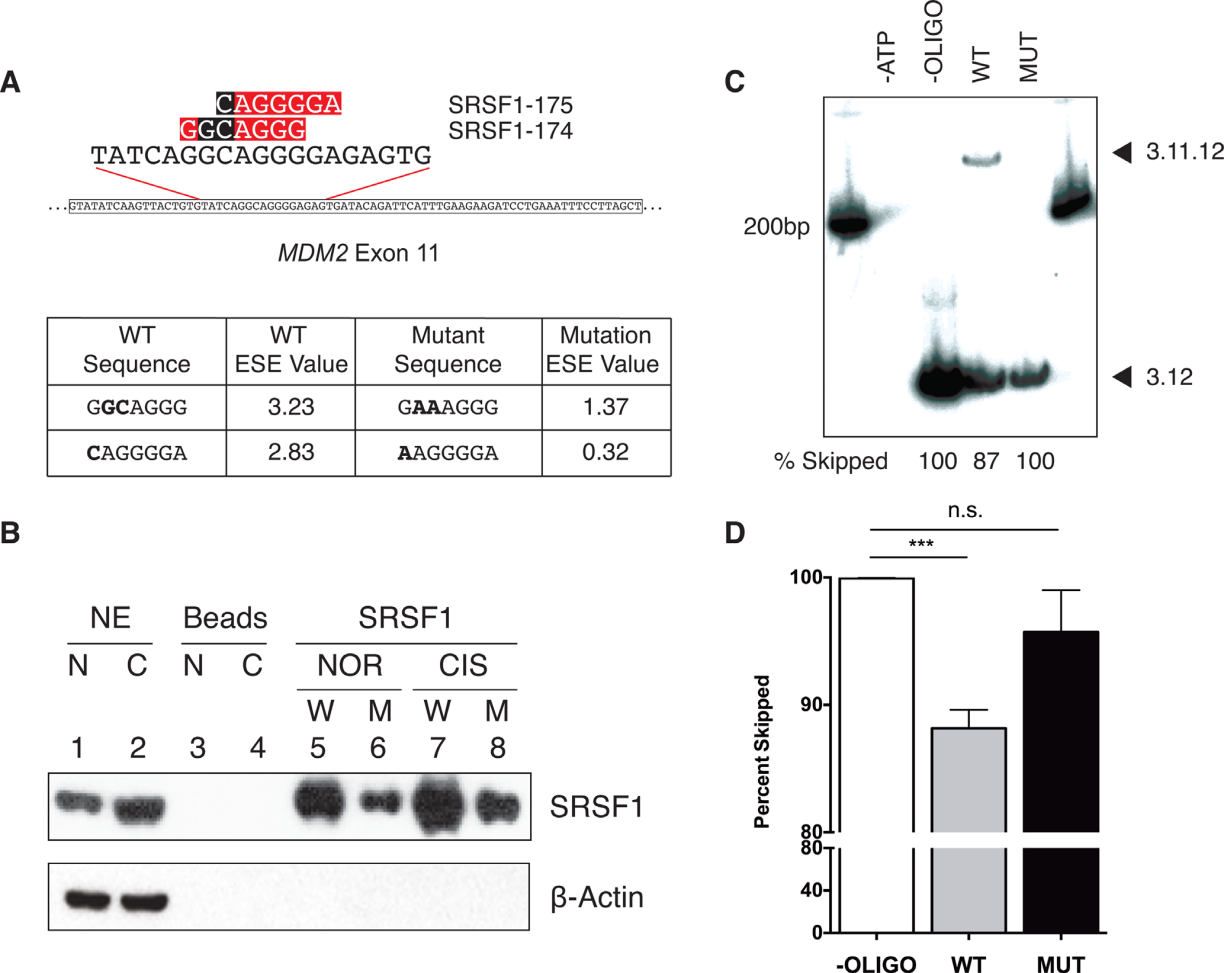
SRSF1 binds predicted exonic splicing enhancer site in *MDM2* exon 11. (**A**) ESEfinder 3.0 (40,41) was used to predict binding sites for SRSF1 (red box). Mutations were made in the *MDM2* 3-11-12*s* minigene (black box) to lower the predicted ESE value. (**B**) Synthesized oligonucleotides, both wild-type and mutant, were conjugated to agarose beads and incubated in normal and cisplatinum-treated HeLa S3 nuclear extract and washed. Proteins were then eluted by heat and subjected to SDS-PAGE analysis to examine differentially-bound proteins. SRSF1 is capable of binding wild-type oligonucleotides (W) in normal (N, NOR) and cisplatinum-damaged (C, CIS) HeLa S3 nuclear extract (NE) and displays diminished binding to mutant (M) oligonucleotides. Representative data of triplicate experiments is shown. (**C**) Splicing reactions were pre-incubated with (WT or MUT) or without oligonucleotides (-OLIGO) in the presence of cisplatinum-damaged HeLa S3 nuclear extract. At 1 h the *MDM2* 3-11-12*s* minigene was added to reactions and spliced for an additional 2 h. RNA was extracted and subjected to a radioactive RT-PCR using a minigene- and gene-specific primer. PCR products were run on a 4% Native-PAGE gel and spliced products were visualized by autoradiography. (**D**) The bar graphs represent the percentage of 3.12 skipped product obtained from three independent experiments under each condition and the error bars represent SEM. The wild-type oligonucleotide binds SRSF1 and rescues the damage-induced alternative splicing of the *MDM2* 3-11-12*s* minigene (*n* = 3).

**Figure 7.**
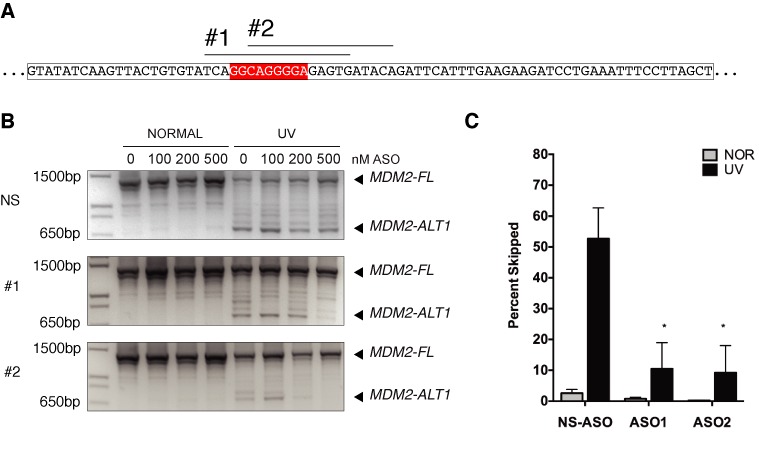
Antisense oligonucleotides (ASOs) targeting SRSF1 binding sites inhibit formation of *MDM2-ALT1*. (**A**) Schematic of SRSF1-specific ASO in *MDM2* exon 11. (**B**) Transfection of ASOs (#1, #2) in MCF-7 cells rescue skipping of endogenous *MDM2* in a dose-dependent manner upon treatment with 50 J/m^2^ ultra-violet (UVC) for 24 h as compared to a non-specific control (NS). RNA was extracted, reverse transcribed, and subjected to a nested PCR. PCR products were separated on a 1.5% agarose gel and spliced products were visualized by UV imaging. Experiments were repeated three times with consistent results. (**C**) The bar graphs represent the percentage of 3.12 skipped product obtained from three independent experiments under each condition and the error bars represent SEM. Transfection of ASO1 and ASO2 were sufficient to ablate induction of endogenous *MDM2-ALT1* under UV-damaged conditions at a concentration at 500 nM, whereas the non-specific ASO had no effect (*n* = 3).

Percentages of full-length and skipped products were quantitated using ImageQuant TL (Version 8.1). Results were plotted in Figures 1–7 and standard error mean (SEM) was used and the significance of the results was assessed using the two-tailed Student's *t*-test using GraphPad Prism (Version 6.0).

### Cell culture, growth and transfection conditions

HeLa and MCF-7 cell lines were maintained in Dulbecco's modified Eagle's medium supplemented with 10% fetal bovine serum (Catalog Number SH3007103) from Thermo Fisher Scientific (Hudson, NH, USA), L-glutamine (Catalog Number MT 25-005 CI) from Corning (Tewksbury, MA, USA) and penicillin/streptomycin (Catalog Number MT 30-001 CI) by Corning (Tewksbury, MA, USA). For transfection of *MDM2* minigenes along with SRSF1 or LacZ overexpression plasmids, cells were seeded to 60% confluency and transfected with either with 0.5 μg of the *MDM2* 3-11-12s wild-type (WT) minigene and 4.5 μg of SRSF1 or LacZ (Figure 5 D–F experiment), or 2.5 μg *MDM2* 3-11-12s WT or 174, 175 mutant minigenes and 2.5 μg of SRSF1 or LacZ (Figure 5A–C experiment) using X-tremeGENE 9 (Catalog Number 06365779001) from Roche (Mannheim, Germany) according to the manufacturer's protocol. For transfection of antisense oligonucleotides (ASOs), MCF-7 cells were seeded to 60% confluency and transfected with Lipofectamine LTX (Catalog 15338-100) from Life Technologies (Carlsbad, CA, USA) according to the manufacturer's protocol. For damage treatment, cells were split into treatment groups (normal, UV, or cisplatinum) 18 h after transfection and treated at 24 h with 50 J/m^2^ ultra-violet (UVC) or 75 μM cisplatinum for 24 h, then harvested for RNA using an RNeasy kit (Catalog 74106) from Qiagen (Valencia, CA, USA) and subjected to RT-PCR using conditions described above; 1 mg/mL stock of cisplatinum (manufactured for Teva Parenteral Medicine Inc., Irvine, CA, USA and obtained from the Nationwide Children's Hospital pharmacy) in sodium chloride solution (pH 3.2–4.4) was used for cisplatinum treatment of cells.

### SRSF1 knockdown

Depletion of SRSF1 was performed using double-stranded siRNAs. The siRNAs targeting human *SRSF1* (*SRSF1* 3′ UTR-siRNA sense, UUGGCAGUAUUGACCUUAUU; *SRSF1* 3′ UTR-siRNA antisense, UAGGUCAAUACUGCCAAUU) or a non-specific siRNA (CTRL sense, AAGGUCCGGCUCCCCCAAAUG; CTRL antisense, CAUUUGGGGGAGCCGGACCUU) were synthesized by Life Technologies (Carlsbad, CA, USA). siRNAs were transfected into MCF-7 cells at a final concentration of 30 nM, mediated by Lipofectamine RNAiMAX from Life Technologies (Carlsbad, CA, USA) for a total of 72 h. At 40 h post-transfection cells were split into normal and UV treatment groups and at 48 h were either treated under normal conditions or exposed to 50 J/m^2^ UVC. Seventy-two hours post-transfection cells were harvested for total RNA using an RNeasy kit (Catalog 74106) from Qiagen (Valencia, CA, USA) and subject to RT-PCR as described above. Protein was also collected as described above to confirm knockdown of SRSF1.

### ASO treatment

2′O-methyl ASOs were generated from Trilink. ASOs specific to *MDM2* exon 11 (#1 ‘GAUUCAGGCAGGGGAGAGUG’, #2 ‘CAGGCAGGGGAGAGUGAUAC’) or a non-specific ASO (‘AUAUAGCGACAGCAUCUUCC’) were transfected into MCF-7 cells using Lipofectamine LTX (Catalog 15338-100) from Life Technologies (Carlsbad, CA, USA) according to the manufacturer's protocol. At 18 h post-transfection cells were split into normal and UV treatment groups and at 24 h were either treated under normal conditions or exposed to 50 J/m^2^ UVC. Twenty-four hours post-treatment cells were harvested for total RNA using an RNeasy kit (Catalog 74106) from Qiagen (Valencia, CA, USA) and subjected to RT-PCR as described above.

## RESULTS

### Minimalized *MDM2* minigene 3-11-12*s* is responsive to stress *in vitro*

In previous work we have shown that *MDM2* minigenes recapitulate the damage-responsive splicing of the endogenous *MDM2* pre-mRNA and thus can be utilized to understand the mechanisms regulating this splicing event (27). We used the previously published damage-responsive minigene 3-11-12 (37) to closely map the *cis* elements that are involved in the regulated splicing of *MDM2*. We engineered a minimal stress-responsive *MDM2* minigene called the 3-11-12*s* minigene. This minigene is comprised of exons 3, 11 and 12 and conserved flanking intronic regions, minimal sequences in the introns, and the core splicing signals of the terminal exons. Specifically, the 3-11-12*s* minigene was created by truncating exons 3 and 12 of the 3-11-12 minigene to retain only 38 nt and 73 nt at their 3′ and 5′ ends, respectively. The upstream chimeric intron (I3/10) of the 3-11-12 minigene was truncated to 72 nt (from 167 nt) and the downstream intron 11 to 147 nt (from 316 nt) in the 3-11-12*s* minigene. Importantly, the internal exon 11 remained intact so splicing regulation could be thoroughly assessed. The 3-11-12*s* minigene, like its parent minigene, is responsive to genotoxic stress *in vitro* (Figure 1A) and *in cellulo* (Figure 4) and excludes internal exon 11 specifically under stress (9.4 ± 4.6% SEM 3.12 product under normal conditions versus 76.2 ± 6.0% SEM under damage conditions), indicating that the minimal sequences included in the 3-11-12*s* minigene are sufficient to recapitulate the stress-induced alternative splicing of *MDM2*. Importantly, the difference in the levels of 3.12 product between normal and cisplatinum-treated conditions was statistically significant (Student's *t*-test, *P* = 0.0009).

### Exon 11 of the *MDM2* 3-11-12*s* minigene is necessary for its genotoxic stress response

To narrow down the *cis* elements that are important for mediating the stress-responsive alternative splicing of the *MDM2* 3-11-*12s* minigene, we employed an intron-exon swap approach between the stress-responsive *MDM2* 3-11-12*s* minigene (Figure 1A) and a non-responsive *p53* 7-8-9 minigene (Figure 1B) (37). Briefly, we generated chimeric minigenes by interchanging the introns and/or the internal exon of the *MDM2* minigene with corresponding regions from the *p53* minigene. In all cases, the 5′ and 3′ splice sites native to the exonic elements were retained (10 nt of the intronic elements flanking the exon and bearing the respective splice sites). These chimeric minigenes were then subjected to *in vitro* splicing in nuclear extracts prepared from normal or cisplatinum-treated HeLa S3 cells and the spliced products were visualized using an RT-PCR approach as described previously (37). The ratio of the skipped product (3.12) to the corresponding full-length spliced product (3.11.12 for the *MDM2* minigene or 3.8.12 for the chimeric minigenes containing the *p53* exon) was determined using the ImageQuant software (Version 8.1) and the percent 3.12 product under each condition is represented graphically and assessed for statistically significant differences between normal and damaged splicing conditions.

When both the introns and exon 11 of the *MDM2* minigene were replaced with introns 7 and 8 and exon 8 of the *p53* minigene the chimeric *MDM2* minigene lost the ability to splice differentially and generated predominantly the exon 11 skipped product (3.12) in both extracts from normal and cisplatinum-treated cells. The splicing of this minigene resulted in the generation of 66.9% (± 6.4% SEM) 3.12 product even in nuclear extracts from normal cells (Figure 2A, Supplementary Figure S1A, lane 4) as opposed to the basal level of 9.4% (± 4.6% SEM) 3.12 product in the WT *MDM2* 3-11-12*s* minigene (observed in three independent experiments; compare Figure 2A to Figure 1A). However, in nuclear extracts from cisplatinum-treated cells the splicing of the chimeric minigene was comparable to the stress-induced splicing of the WT *MDM2* minigene and generated 78.9% (± 7.4% SEM) of the 3.12 skipped product across three separate trials (compare Figure 2A, Supplementary Figure S1A, lane 5, to Figure S1A). Moreover, the difference in the percent 3.12 splicing of the chimeric minigene between normal and cisplatinum-damaged conditions was not statistically significant (Student's *t*-test *P* = 0.2883, Figure 2A), unlike the WT *MDM2* 3-11-12*s* minigene. This indicates that the elements contained within the introns and/or the internal exon 11 of the *MDM2* 3-11-12*s* minigene are necessary for the damage-specific response and their loss resulted in the formation of a steady expression of this 3.12 product even under normal conditions (Figure 2A, F, Supplementary Figure S1A, lanes 4–5).

We next removed either the upstream (I3/10) (Figure 2B, Supplementary Figure S1A, lanes 12–13) or downstream (I11) (Figure 2C, Supplementary Figure S1A, lanes 6–7) or both introns (Figure 2D, Supplementary Figure S1A, lanes 8–9) from the *MDM2* minigene and replaced them with the corresponding introns from the non-responsive *p53* minigene (5′ and 3′ splice sites in these constructs were those native to the exons of the respective minigene and not from the introns being inserted). These chimeric *MDM2* minigenes retained the damage response and showed a statistically significant increase in percent 3.12 skipped product in nuclear extract from cisplatinum-damaged cells (an average of 67.5% for all three chimeric minigenes in three separate experiments) compared to the nuclear extract from normal cells (an average of 32.1%; Figure 2B, C, D, F, Supplementary Figure S1A, lanes 12–13, 6–7, 8–9). This behavior was comparable to the damage-responsive splicing of the WT *MDM2* 3-11-12*s* minigene, although there was a slight increase in the baseline percent skipped 3.12 product in the normal nuclear extract (compare Figure 1A, Supplementary Figure S1A, lane 2 to Figure 2B, C, D, F, Supplementary Figure S1A, lanes 12, 6, 8). However, when exon 11 of the *MDM2* minigene was removed and replaced with exon 8 of the *p53* minigene the chimeric *MDM2* minigene failed to show the damage-responsive splicing ratio change (Figure 2E, F, Supplementary Figure S1A, lanes 10–11). Indeed, the percent 3.12 skipped product obtained when this minigene was spliced in nuclear extracts from normal cells (16.9 ± 11.2% SEM) and the percent 3.12 obtained from splicing in nuclear extracts from cisplatinum-damaged cells (17.9 ± 2.8% SEM) were not significantly different (Student's *t*-test, *P* = 0.9344). Together, these data indicate that exon 11 of the *MDM2* minigene contains important elements that regulate its damage-responsive alternative splicing.

### Exon 11 of the *MDM2* 3-11-12*s* minigene is necessary and sufficient to sustain genotoxic stress response in a heterologous context

We then constructed reciprocal chimeras of the *p53* minigene, which normally does not show splicing changes in response to stress (36.1 ± 4.8% SEM of the 7.9 skipped product under normal or 40.1 ± 3.4% SEM under cisplatinum-damaged conditions; Figure 1B). For these constructs, we replaced native elements of the *p53* minigene with the corresponding intronic or exonic elements of the *MDM2* minigene. When exon 8 of the *p53* minigene and its flanking introns were replaced with both flanking introns and exon 11 of the *MDM2* minigene the chimeric *p53* minigene exhibited damage-responsive alternative splicing similar to the WT *MDM2* minigene (percentage of 7.9 spliced product was 12.6 ± 4.2% SEM under normal and 75.1 ± 5.5% SEM under cisplatinum-damaged conditions, *P* = 0.0008 with Student's *t*-test) (Figure 3A, D, Supplementary Figure S2A, lanes 4–5). This indicates that the *cis* elements contained within the *MDM2* minigene's internal exon and introns are sufficient to facilitate damage-specific alternative splicing in the heterologous *p53* minigene system. The chimeras in which intron 7 of the *p53* minigene was removed either by itself or in conjunction with the downstream intron 8 failed to splice at all in nuclear extracts from both normal and damage-treated cells as only the unspliced minigene transcripts were detected after RT-PCR (Figure 3D, Supplementary Figure S2A, lanes 8–9 and 12–13). When intron 8 of the *p53* minigene was replaced with intron 11 of the *MDM2* minigene there was a modest increase in the skipped 7.9 product in response to damage (40.7 ± 8.9% SEM under cisplatinum-damaged compared to 25.1 ± 2.5% SEM under normal conditions), although this change was not statistically significant (Student's *t*-test *P* = 0.1694) (Figure 3B, D, Supplementary Figure S2A, lanes 6–7). Strikingly, when exon 11 of the *MDM2* minigene was inserted in the *p53* minigene (*MDM2* exon 11 was placed in the heterologous *p53* minigene with its own exon 11 5′ and 3′ splice sites native to *MDM2*) in the place of the native *p53* exon 8, the chimeric minigene responded to cisplatinum damage unlike the WT *p53* minigene when spliced in nuclear extracts from stressed cells. Indeed, the percentage of the 7.9 skipped product increased from 32.7% (± 5.4% SEM) in nuclear extract from normal cells to 71.1% (± 5.9% SEM) in nuclear extracts from cisplatinum-treated cells (Figure 3C, D, Supplementary Figure S2A, lanes 10–12) and this difference was found to be statistically significant (Student's *t*-test, *P* = 0.0084). In short, the chimeric 7-11-9 *p53* minigene behaved like the WT *MDM2* 3-11-12*s* minigene in response to damage indicating that *MDM2* exon 11 is sufficient to confer damage response in a heterologous minigene context.

### SRSF1 is a negative regulator of *MDM2* alternative splicing

To identify splicing factors that may be responsible for the damage-responsive alternative splicing of the *MDM2* minigene we performed bioinformatics analysis of exonic splicing enhancers (ESEs) present in exon 11. Using a SELEX-based (systematic evolution of ligands by exponential enrichment) program called ESEfinder 3.0, which takes consensus-binding motifs for SR proteins derived from selective enrichment of 20 nt random sequences for the splicing of a minigene in S100 extract supplemented with individual SR proteins, we entered the sequence of our *MDM2* minigene to examine predicted binding sites for SR proteins (40,41). Among the top hits was a site in *MDM2* exon 3 and an overlapping pair of SRSF1 binding sites in exon 11, all of which were conserved between mouse and human *MDM2*. We then performed point mutations in our *MDM2* minigene to disrupt the binding affinity of SRSF1 for its predicted sites (Supplementary Figure S3A). Importantly, we made precise mutations that maintained other binding sites for overlapping bioinformatically-predicted factors SRSF2 (SC35), SRSF5 (SRp40) and SRSF6 (SRp55). In the case of the pair of SRSF1 binding sites in exon 11, a single mutation was not sufficient to disrupt both SRSF1 binding sites, so a double mutant, SRSF1-174, 175, was created. The strength of each splicing enhancer site corresponds to a scale in which a higher numeric matrix score indicates greater predicted binding strength. The mutations made in SRSF1-48 and SRSF1-174, 175 significantly lowered the predicted ESE value from 3.05 to 0.74 and 3.23 to 1.37, respectively (Supplementary Figure S3A). We then performed site-directed mutagenesis of these sites on our *MDM2* minigene to assess the effects of these mutations on splicing.

We examined the splicing of the WT and SRSF1 mutant *MDM2* minigenes *in vivo* in HeLa and MCF-7 cells. These cell lines were chosen for their relative ease of transfection and ability to tolerate genotoxic stress. The splicing patterns of the WT and the SRSF1 mutant minigenes were compared under the different conditions. Although the mutation at the SRSF1-48 site was predicted to disrupt the ESE in exon 3 (the matrix score was lowered for SRSF1-48), we observed that the corresponding mutant minigene (15.890 ± 5.683% SEM NOR, 49.220 ± 1.265% SEM UV) did not show altered splicing compared to WT (19.050 ± 6.466% NOR, ± 48.630 ± 1.860% UV) under both the normal (*P* = 0.7317) and UV-treated conditions (*P* = 0.8049) (Supplementary Figure S3B). However, mutation of the SRSF1 sites in *MDM2* exon 11 (174, 175 mutant minigene) eliminated the damage-responsive exon 11 skipping upon UV (MCF-7 0.270 ± 0.01958% SEM, HeLa 4.933 ± 0.3093% SEM) and cisplatinum treatment (MCF-7 2.235 ± 0.4246% SEM, HeLa 1.800 ± 0.6848% SEM) compared to the skipping of the WT minigene under UV and (MCF-7 31.200 ± 2.140% SEM, HeLa 39.227 ± 2.819% SEM) cisplatinum (MCF-7 33.935 CIS ± 1.709% SEM, HeLa 27.330 ± 0.7490% SEM) treatments (Figure 4). The decrease in exon 11 skipping observed in the SRSF1 174, 175 mutant minigene under normal and damaged conditions was statistically significant when compared to the WT minigene (Student's *t*-test *P* < 0.0001).

### SRSF1 overexpression induces exclusion of *MDM2* exon 11

To determine whether SRSF1 acts as a regulator of *MDM2* alternative splicing we overexpressed a T7-tagged SRSF1 construct or a negative control, LacZ and the WT 3-11-12*s* minigene in MCF-7 cells. Compared to LacZ (8.487 ± 1.149% SEM) SRSF1 overexpression (39.700 ± 6.322% SEM) significantly (*P* = 0.0007) induced skipping of exon 11 in the WT minigene even in the absence of genotoxic stress (Figure 5A–C). Similarly under UV-damaged conditions, overexpression of SRSF1 induced higher levels of 3.12 skipped product (62.020 ± 5.016% SEM) compared to LacZ overexpression (28.020 ± 1.722% SEM) (*P* = 0.0002) (Figure 5A–C). A similar experiment was performed using the 3-11-12*s* mutant minigene, for which the SRSF1-174, 175 sites were mutated. The ability of SRSF1 overexpression to induce exclusion of exon 11 was reduced (3.550% ± 0.8709 SEM) when coexpressed with the SRSF1-174, 175 mutant minigene when compared to the WT minigene (16.390% ± 1.675 SEM) (Figure 5D–F). Overexpression of MYC-LacZ and T7-SRSF1 were confirmed by immunoblotting (Figure 5C and F). These results suggest a negative role for SRSF1 in the regulation of *MDM2* splicing.

To confirm that the effect of SRSF1 was not a non-specific effect due to the protein's ability to bind RNA we tested additional RNA binding proteins whose binding was not predicted using ESEfinder 3.0. To this end we overexpressed a panel of hnRNPs (D, F, L, R, and U) in MCF-7 cells and assessed their function on the splicing of the *MDM2* 3-11-12*s* minigene under normal and UV-treated conditions. We observed that the splicing patterns of the *MDM2* 3-11-12*s* minigene did not show any significant differences between LacZ and hnRNP overexpression under both normal and damaged conditions (Supplementary Figure S4A and B). Overexpression of LacZ and the individual hnRNPs was confirmed by immunoblotting (Supplementary Figure S4C).

### SRSF1 knockdown rescues damage-induced skipping of *MDM2*

Next we examined the effects of SRSF1 knockdown on the ability of genotoxic stress to induce *MDM2-ALT1*. To this end we transfected MCF-7 cells with a non-specific (CTRL) or *SRSF1*-specific siRNA (SRSF1). We observed that siRNA-mediated knockdown of SRSF1 resulted in approximately a six-fold decrease (*P* = 0.0082) in the percentage of *MDM2-ALT1* (endogenous 3.12 skipped product) induced under UV treatment (9.297 ± 4.159% SEM) when compared to non-specific siRNA-transfected cells (60.950 ± 9.757% SEM) (Figure 5G and H). We confirmed efficient knockdown (85–95%) of SRSF1 by immunoblotting (Figure 5I). Taken together, these data further support SRSF1 as a negative regulator of *MDM2* alternative splicing.

We observe an increase in relative SRSF1 protein levels under UV treatment (Figure 5I, lane 3) compared to normal conditions (Figure 5I, lane 1). To investigate the UV-induced upregulation of SRSF1, we examined its transcript levels at several time points over 24 h of UV irradiation using qRT-PCR (Supplementary Figure S5A). As a positive control we examined the levels p53-responsive *CDKN1A* (cell-cycle regulator p21), whose expression is upregulated at both transcript and protein levels under conditions of genotoxic stress (42). As expected, *CDKN1A* transcript levels increased upon UV treatment (Supplementary Figure S5A). However in the case of SRSF1, we did not observe an increase in transcript levels (Supplementary Figure S5A). Rather, we observe a decrease in *SRSF1* transcripts over the course of 24 h of UV treatment (Supplementary Figure S5A), a phenomenon that is consistent with a general inhibition of RNA synthesis under DNA-damaging conditions (43). However, another means of regulating *SRSF1* levels is via its alternative splicing in the 3′ UTR and six major splice variants have been characterized, of which only isoforms I and II can generate full-length protein (44). When we examined the relative levels of the various splice forms of *SRSF1* between normal and DNA damage conditions, we observed a significant increase (*P* = 0.0005) in the levels of the productive isoforms I and II under UV treatment (64.667 isoform I/II ± 2.028% SEM) as compared to normal treatment (36.333 isoform I/II ± 1.856% SEM) with a concomitant decrease in expression of isoforms III to VI (Supplementary Figure S5B). This raises the possibility that upregulation of the productive splice forms I and II under UV contributes to the observed increase in SRSF1 levels.

### SRSF1 binds exonic splicing enhancer elements in *MDM2* exon 11

To determine whether SRSF1 acts as a regulator of *MDM2* alternative splicing via direct binding to exon 11 we performed *in vitro* binding studies. We synthesized both wild-type and mutant oligonucleotides encompassing the binding site in exon 11 and tested their ability to bind or pull down SRSF1 in splicing-competent nuclear extracts (Figure 6A). We performed an *in vitro* RNA oligonucleotide pull down using wild-type and mutant oligonucleotides in nuclear extracts from both normal and cisplatinum-treated HeLa S3 cells. SRSF1 showed increased binding to the wild-type oligonucleotide under cisplatinum-damaged conditions as compared to normal conditions (Figure 6B, Lane 5, 7), consistent with the increased levels of SRSF1 in the cisplatinum-treated nuclear extract (Figure 6B, Lane 1, 2). Importantly, SRSF1 showed decreased binding to the mutant oligonucleotide both under normal and cisplatinum-damaged conditions (Figure 6B, Lanes 6 and 8) indicating that mutation of these sites in exon 11 attenuates SRSF1 binding. Furthermore, we observed that a molar excess of the wild-type exon 11 oligonucleotide, but not the 174, 175 mutant was able to successfully compete with and alter the splicing of the WT *MDM2* 3-11-12*s* minigene in nuclear extracts from cisplatinum-treated Hela S3 cells (compare percent 3.12 skipped product in the absence of competing oligonucleotides [99.940 ± 0.023% SEM] and in the presence of WT [88.160 ± 1.455% SEM, (*P* = 0.0002)] or mutant [95.720 ± 3.282% MUT (*P* = 0.1852)] oligo; Figure 6C and D). Taken together these results indicate that SRSF1 binds *MDM2* exon 11 at the 174, 175 site and regulates the damage-induced alternative splicing. Mutations at this site that inhibit SRSF1 binding also abrogate the stress-specific exclusion of exon 11.

### ASOs modulate endogenous *MDM2* alternative splicing under genotoxic stress

To investigate the importance of the SRSF1 binding elements in *MDM2* exon 11 in the regulation of endogenous *MDM2* splicing, we designed 2′O-methyl antisense oligonucleotides (ASOs) targeting this region. We predicted that binding of the ASOs to exon 11 SRSF1 sites via complementary nucleotide base paring would occlude binding of the SRSF1 protein (Figure 7A). To test this, we transfected MCF-7 cells with exon 11 SRSF1 ASOs (ASO1 and ASO2) and a non-specific control ASO (NS-ASO). At the highest doses (500 nM) both ASO1 (10.480 ± 8.503% ASO1) and ASO2 (9.253 ± 8.772% ASO2) targeting the SRSF1 sites in exon 11 ablated the formation of endogenous *MDM2-ALT1* under UV-damaged conditions (Figure 7B) and this difference in induction was statistically significant (Figure 7C, ASO1 *P* = 0.0322 and ASO2 *P* = 0.0307). However, the non-specific ASO (NS) had no effect and *MDM2-ALT1* transcripts were induced under UV-treated conditions at all doses of the NS-ASO (Figure 7B).

### SRSF1 is overexpressed in rhabdomyosarcoma patient samples

**Figure 8.**
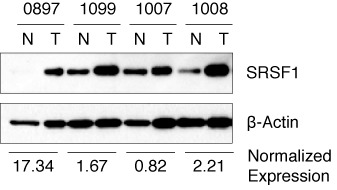
SRSF1 is upregulated in rhabdomyosarcoma (RMS) tumor tissues. Frozen rhabdomyosarcoma patient samples and normal tissue-matched control were homogenized and extracted for protein. Protein lysates were run on a 10% SDS-PAGE gel and probed with SRSF1 and β-Actin antibodies to confirm protein levels. SRSF1 is upregulated in tumor tissues of RMS patients compared to their normal tissue-matched controls. Normalized expression values for tumor (T) samples compared to normal muscle (N) are depicted below the graph.

*MDM2-ALT1* expression is observed in several cancer types including breast (33,34), colon (35) and glioblastoma (45). Additionally, we have shown that *MDM2-ALT1* is expressed in over 85% alveolar and 70% embryonal rhabdomyosarcoma (RMS) tumors and that its expression is correlated with high-grade metastatic disease, irrespective of histological subtype (5). To examine the relationship between the perturbed splicing of *MDM2* and the expression of SRSF1 we examined a panel of four RMS tumors that express *MDM2-ALT1* constitutively and for which matched normal tissues were available. We observed elevated SRSF1 levels in three of the four tumor samples compared to their corresponding normal tissue-matched controls (Figure 8). Though the number of samples available with matched normal controls was small, the elevated SRSF1 expression in tumor samples correlated with our finding that overexpression of SRSF1 induces *MDM2-ALT1*.

## DISCUSSION

DNA damage-induced alternative splicing of *MDM2* is observed in both human and mouse transcripts (46). Additionally, both human and mouse *Mdm2* possess conserved SR protein binding sites in exon 11 suggesting that the alternative splicing of *MDM2* could be an important evolutionarily conserved mechanism for the titration of MDM2 levels under stress. Furthermore, functional studies have revealed a role for the stress-inducible splice forms of *MDM2* in cancer, underscoring the importance of this splicing event and the necessity to gain an understanding of the mechanisms involved in the damage-responsive splicing of *MDM2*. Using a novel damage-inducible *in vitro* splicing system we have previously shown that intron 11 of *MDM2* contains conserved positive elements that are primarily needed for the efficient full-length splicing of *MDM2* (37,38). However, the factors governing its damage-responsive alternative splicing still remained to be elucidated.

In this study, we have made use of a minimal 3-11-12*s* minigene system to identify the *cis* splicing regulatory elements and the *trans* factors that directly mediate the damage-induced skipping of *MDM2* exon 11. Using an intron-exon swap approach between the stress-responsive 3-11-12*s* and a non stress-responsive *p53* minigene (37) we demonstrate that exon 11 of *MDM2* contains elements that are not only necessary, but also sufficient to regulate its damage-specific alternative splicing even in a heterologous *p53* minigene context (Figures 1, 2 and 3). Moreover, this effect is independent of both the introns (upstream intron 3/10 and downstream intron 11) of the minimal 3-11-12*s* minigene (Figures 2 and 3). Interestingly, in a previous study we observed that the splicing regulation of a larger version of the 3-11-12 *MDM2* minigene containing additional positive acting elements in intron 11 (absent in the 3-11-12*s* minigene of the present study) (38) was dependent upon intron 11 (37). It is likely then that the shortened intron 11 of the minimal 3-11-12*s* minigene lacks the positive acting elements and also the counter-balancing negative elements, thereby facilitating neutralization and eliminating the requirement for intronic regulatory elements. However, it should be noted that intron 11, irrespective of the *MDM2* minigene that it was derived from, was insufficient to confer damage-responsive alternative splicing in the heterologous *p53* minigene context (Figure 3 and (37)). Hence, in the context of endogenous *MDM2* pre-mRNA it is feasible to envision a scenario in which splicing regulation under normal and DNA-damaged conditions is mediated by complex interactions between the intronic and exonic *cis*-acting elements.

### SRSF1-mediated splicing repression

In the present case, we identify a conserved exonic splicing silencer (ESS) element in exon 11 whose disruption results in the loss of exon 11 skipping in response to DNA damage. Furthermore, we present evidence that SRSF1 binds this site and acts as a negative regulator of *MDM2* splicing. Although canonically considered a splicing enhancer (ESE), SRSF1 has also been shown to act as a negative regulator of splicing in certain contexts (46–54). Well-known examples of SRSF1-mediated exon exclusion include the splicing of *RONΔ11*, a pro-oncogenic isoform of the Tyrosine kinase receptor *RON*,and the exon 9-excluded form of *CFTR*. In the case of *RON* the skipping of exon 11 is dependent on the binding of SRSF1 to ESE and ESS elements in the adjacent exon 12 (55). This generates *RONΔ11* that promotes cellular invasion and motility (50,55). The best-characterized mechanism for SRSF1-mediated exon skipping is the binding of SRSF1 to a silencer motif in the intron downstream of *CFTR* exon 9, which allows the assembly of splicing machinery on a decoy exon thereby repressing the functional splicing signals in exon 9 (52). However, the exact nature of this repression remains unclear.

Thus far, in the best-characterized examples of SRSF1-mediated splicing repression this SR protein acts via intronic silencer elements (ISS) (48,52) or enhancer elements located in the exons (ESE) flanking the regulated exon (55). Moreover, studies have shown that classical SR protein-binding ESE sequences when inserted into intronic locations can prevent splicing to the downstream 3' splice site thus acting as repressors of splicing (56). In a converse scenario, when ISS elements bound by the SRSF10 (TRA2B) are relocated to an exon, they act as ESEs and favor exon inclusion (57).

In the case of *MDM2*, we report a unique instance wherein the damage-specific skipping of exon 11 is mediated by SRSF1 via a predicted ESE element located in the regulated exon itself. It is possible that the location of the element is responsible for directing the functionality of its SR protein binding partner. Indeed, position-dependent effects have been reported for the activity of exonic splicing regulatory elements that potentially shift the nature of the SR protein-mediated splicing regulation of alternative versus constitutive exons (58). Additionally, complex evolutionary relationships exist between the exonic splicing regulatory elements (ESRs) and the alternatively-spliced or regulated exons whose splicing they control. These involve strength of the 5′ and 3′ splice sites flanking regulated exons, conservation, location, and abundance of the ESRs and various other factors that blur the functional distinction between splicing enhancers and splicing silencer elements on alternatively-spliced exons (58). These studies argue that SRSF1-mediated exon inclusion or exclusion relies on the contextual information of the surrounding exonic and intronic regions.

Moreover, transcriptome-wide analyses have correlated regulated exons with higher occurrence of ESS elements compared to constitutive exons that present with an abundance of ESE elements (59,60). This is concordant with studies showing that majority of the alternative splicing in metazoans represents exon skipping events (61). Taken together, these results suggest that exon 11 and potentially the other exons of *MDM2* that are skipped in response to stress harbor ESR elements whose location dictates ESS or ESE functionality and modulates the role of the *trans* protein factors binding them. However, more detailed computational analyses of the ESRs of *MDM2* exons in relation to their splice site strengths, sequence conservation, and *trans* factor binding site predictions coupled with experimental validation of the ESR functions are required to test this possibility.

Notably, SRSF1 binds the element on exon 11 both under normal and damaged conditions (Figure 6). One possibility is that SRSF1 binding to exon 11 is non-functional in normal conditions and even serves to mask the ESS element. Another possibility is that SRSF1 binding to exon 11 under normal conditions serves as a splicing enhancer. However, the mutant *MDM2* minigenes unable to bind SRSF1 do not show even baseline exon skipping under normal conditions indicating that this possibility may not be true (Figure 4). Post-translational modifications including phosphorylation of SR proteins have been shown to modulate their catalytic activity (9,62–64), suggesting that differential phosphorylation of SRSF1 could account for its activity under normal and damaged conditions. We found that this was also not the case because we have observed no differences in the migration of SRSF1 between nuclear extracts from normal and cisplatinum-treated HeLa S3 cells that were either untreated or incubated with calf intestinal phosphatase (38).

What we did observe was an increase in the levels of SRSF1 in nuclear extracts from cisplatinum-treated Hela S3 cells compared to nuclear extracts from normal cells. Concordantly, we find increased binding of SRSF1 to exon 11 under DNA damage compared to normal conditions (Figure 6, Lane 7). Hence, we propose a critical level of SRSF1 binding is necessary to cause repression of exon 11 splicing as seen under genotoxic stress. It is possible that under these conditions SRSF1 binding overrides the influence of other positive elements and *trans* factors and precludes the recognition of the flanking 5′ and 3′ splice sites and consequently the definition of exon 11 by the spliceosomal complex. Additionally, the differential binding of SRSF1 under DNA damage raises the intriguing possibility of crosstalk with other SR protein factors. For instance, the binding of other SR proteins and *trans* factors to ESRs adjacent to the SRSF1 site could cause their functional interaction with SRSF1 in a yin and yang fashion that mediates exon 11 inclusion under normal conditions and facilitates its exclusion in response to stress.

### Impact on cancer

SRSF1 is located on chromosome 17 and is a commonly amplified region in breast cancer, correlating with poor prognosis (65). SRSF1 regulates the alternative splicing of several tumor suppressor genes, kinases and kinase receptors, all of which generate oncogenic isoforms (19). Furthermore Karni *et al*. have demonstrated that slight SRSF1 overexpression is capable of inducing cellular transformation in immortalized rodent fibroblasts *in vitro* as well as inducing sarcoma formation in nude mice (19). As changes in alternative splicing have been shown to be important for the neoplastic phenotype, the global patterns of alternative splicing upon SRSF1 upregulation are important to understand. Recently, de Miguel *et al*. demonstrated that over 20 transcripts were regulated by SRSF1 in lung cancers. For example, siRNA-mediated knockdown of SRSF1 in this study prevented the inclusion of a lung carcinoma-associated exon in the transcript *PRRC2C* and significantly reduced cell growth (47). Moreover, *SRSF1* has been demonstrated to be a direct transcriptional target of the oncogene c-Myc further cementing the role of SRSF1 in oncogenesis (66).

In our study, we have demonstrated that SRSF1 is capable of regulating the stress-induced alternative splicing of the oncogene *MDM2*. Indeed, we show that pediatric rhabdomyosarcoma tumors spontaneously expressing *MDM2-ALT1* (5) also show elevated levels of SRSF1 compared to matched normal muscle tissue (Figure 8). This is important because *MDM2-ALT1*, the alternative splice variant of *MDM2* that is predominantly generated in response to DNA damage, is also strongly associated with several cancer types (31–36). *In vitro* studies have demonstrated the tumorigenic potential of MDM2-ALT1 (5,29,36). *In vivo*, the mouse homolog Mdm2-b has been shown to lead to tumorigenesis in a syngeneic mouse model, while an MDM2-ALT1-like protein accelerated lymphomagenesis in Eμ-Myc mice (29,67). Paradoxically, MDM2-ALT1 expression results in the upregulation of the tumor suppressor p53 and the activation of a subset of its transcriptional targets. This is because MDM2-ALT1 lacks the p53-binding domain and is therefore incapable of binding and negatively regulating p53. Moreover, it functions as a dominant negative protein by dimerizing with (via the RING domain) and sequestering full-length MDM2 (10,26,30,39,68). Interestingly, a recent study demonstrated that in the context of tumors presenting with mutant gain-of-function p53, the expression of MDM2-ALT1 can inhibit the degradation of mut-p53 by interfering with the function of full-length MDM2 leading to accumulation of mutant p53 in tumor cells (68). However, several tumor types including rhabdomyosarcomas that present with *MDM2-ALT1* have predominantly wild-type p53 (5,45). This indicates that perhaps some effects of *MDM2* alternative splicing are p53-independent. It is therefore unclear whether MDM2-ALT1 is capable of promoting transformation through other p53 family members such as p63 and p73, or other pathways entirely.

### Opportunity for therapeutic intervention

Because the chief function of full-length MDM2 is to promote the degradation of p53, modulating the splicing of *MDM2* to yield splice variants incapable of such regulation could prove to be a valuable strategy to manipulate p53 levels. For example, in tumors presenting with mutant p53 and *MDM2-ALT1* (68), blocking the SRSF1 binding sites in exon 11 would facilitate the expression of more full-length transcripts and consequently more functional full-length MDM2 protein to degrade mut-p53. We show that treatment with ASOs to block SRSF1 binding sites in *MDM2* exon 11 promotes a decrease in the *MDM2-ALT1* alternatively-spliced transcript under stress. These results demonstrate the efficacy of the use of ASOs for targeting this site for splicing modulation of *MDM2*.

Importantly, it is likely that there are positive elements that antagonize the regulation of SRSF1 in *MDM2* exon 11. Once these are identified, they could similarly be targeted to generate more *MDM2-ALT1* and reactivate wild-type p53 (MDM2-ALT1 stabilizes p53 when by opposing full-length MDM2) (10,30,39), thus inducing massive apoptosis to combat the action of other constitutively-active oncogenes. In short, controlling the ratio of the *MDM2* splice isoforms using ASOs is an attractive strategy to control p53 levels, whether wild-type or mutant in cancer cells.

In the present study we have provided evidence that overexpression and increased binding of SRSF1 to *MDM2* exon 11 are sufficient to drive the expression *MDM2-ALT1*. This is the first description of a molecular mechanism underpinning the alternative splicing of *MDM2* under damage, raising the possibility that persistent *MDM2-ALT1* splicing observed in cancers is regulated by the same means. By understanding the molecular mechanisms regulating *MDM2* splicing in response to damage and potentially in cancer we have paved the way for the development of novel splice modulation strategies for adjusting *MDM2* levels in cancers with elevated *MDM2-ALT1* and SRSF1 expression. Future studies to identify other modifiers of *MDM2* splicing will enable a comprehensive understanding of stress, cancer-induced splicing, and the design of specific splicing modulation strategies.

## SUPPLEMENTARY DATA

Supplementary Data are available at NAR Online.

SUPPLEMENTARY DATA
