# Supplementary material for: Splicing factor SRSF1 negatively regulates alternative splicing of MDM2 under damage
Source: Nucleic Acids Res. 2015 Apr 6;43(8):4202–18. doi: 10.1093/nar/gkv223 (PMC4417157; doi:10.1093/nar/gkv223)
Supplement: SUPPLEMENTARY DATA [file supp_43_8_4202__index.html]

Splicing factor SRSF1 negatively regulates alternative splicing of MDM2 under damage — Splicing factor SRSF1 negatively regulates alternative splicing of MDM2 under damage — SUPPLEMENTARY DATA 

# Splicing factor SRSF1 negatively regulates alternative splicing of *MDM2* under damage

## SUPPLEMENTARY DATA

**Files in this Data Supplement:**

- SUPPLEMENTARY DATA
- SUPPLEMENTARY DATA
