# Supplementary material for: Splicing factor SRSF1 negatively regulates alternative splicing of MDM2 under damage
Source: Nucleic Acids Res. 2015 Apr 6;43(8):4202–18. doi: 10.1093/nar/gkv223 (PMC4417157; doi:10.1093/nar/gkv223)
Supplement: SUPPLEMENTARY DATA [file supp_gkv223_nar-03002-a-2014-File010.docx]

**Supplemental Figure 1: Loss of *MDM2* exon 11 abolishes stress-responsive alternative splicing of the *MDM2* minigene.** Chimeric *MDM2* minigenes were created by replacing the introns and/or internal exon of *MDM2* with corresponding regions from the non stress-responsive *p53* minigene. These minigenes were then spliced *in vitro* in nuclear extracts prepared from normal (N) and cisplatinum (C) treated cells. RNA was extracted using phenol/chloroform, reversed transcribed, and subjected to a 25-cycle PCR using γ-^32^P-radioactively-labeled Flag primer and gene-specific reverse primers. **(a)** Full-size autoradiograph of representative splicing reactions from *MDM2* chimeric minigene +ATP reactions. *Indicates non-specific band also seen in -ATP controls. ^Indicates probable PCR degradation products. Sizes of each linear spliced and unspliced products are depicted in the table. **(b)** Full-size autoradiograph of representative splicing reactions from *MDM2* chimeric minigene -ATP reactions and blank (B) reaction. Sizes of each linear spliced and unspliced products are depicted in the table.

**Supplemental Figure 2: *MDM2* exon 11 is sufficient to regulate stress-responsive splicing in the heterologous *p53* minigene context.** Chimeric *p53* minigenes were created by replacing the introns and/or internal exon of *p53* with corresponding regions from the stress-responsive *MDM2* minigene. These minigenes were then spliced *in vitro* in nuclear extracts prepared from normal (N) and cisplatinum (C) treated cells. RNA was extracted using phenol/chloroform, reversed transcribed, and subjected to a 25-cycle PCR using γ-^32^P-radioactively-labeled Flag primer and gene-specific reverse primers. **(a)** Full-size autoradiograph of representative splicing reactions from *p53* chimeric minigene +ATP reactions. *Indicates non-specific band also seen in -ATP controls. ^Indicates probable PCR degradation products. Sizes of each linear spliced and unspliced products are depicted in the table. **(b)** Full-size autoradiograph of representative splicing reactions from *p53* chimeric minigene -ATP reactions and blank (B) reaction. Sizes of each linear spliced and unspliced products are depicted in the table.

**Supplemental Figure 3: Mutation of the SRSF1 binding site in *MDM2* Exon 3 does not affect splicing of the *MDM2* 3-11-12s minigene. (a)** Schematic of *MDM2* 3-11-12s minigene with predicted SRSF1 binding sites in exon 3 and exon 11 (red boxes) and subsequent mutations made (black boxes). A table of wild-type and mutant sequences of SRSF1 sites and relative ESEfinder 3.0 matrix scores. **(b)** *MDM2* minigenes were transfected into MCF-7 cells and treated under normal, 50 J/m^2^ ultra-violet (UVC) conditions for 24 hours. RNA was extracted and subjected to RT-PCR using a minigene and gene-specific primer. PCR products were separated on a 1.5% agarose gel and spliced products were visualized by UV imaging. The SRSF1-48 mutant does not lose damage-inducible alternative splicing of exon 11 (n=3).

**Supplemental Figure 4: Expression of hnRNPs does not affect splicing of the *MDM2* 3-11-12s minigene. (a)** The *MDM2* 3-11-12s minigene and LacZ or hnRNP constructs were cotransfected in MCF-7 cells for 24 hours and then treated under normal or 50 J/m^2^ ultra-violet (UVC) conditions for an additional 24 hours. RNA was extracted and subjected to a RT-PCR using a minigene and gene-specific primer. PCR products were separated on a 1.5% agarose gel and spliced products were visualized by UV imaging. **(b)** The bar graphs represent the percentage of 3.12 skipped product obtained from three independent experiments under each condition and the error bars represent standard error mean (SEM). The splicing of the *MDM2* 3-11-12s minigene was unaffected by hnRNP D, hnRNP F, hnRNP L, hnRNP R, or hnRNP U compared to LacZ overexpression. **(c)** Protein lysates were run on a 10% SDS-PAGE gel and probed with GFP, C-MYC, HA, Flag and β-Actin antibodies to confirm protein overexpression.

**Supplemental Figure 5: *SRSF1* transcription is downregulated and isoforms I/II are upregulated in response to UV treatment. (a)** MCF-7 cells were treated under 50 J/m^2^ ultra-violet (UVC) conditions for the time indicated and subjected to qPCR for *SRSF1* and *CDKN1A*. The bar graphs represents the percentage of relative expression values compared to no treatment (0h) obtained from three independent experiments under each condition and the error bars represent standard error mean (SEM). **(b)** MCF-7 cells were treated under normal or 50 J/m^2^ ultra-violet (UVC) for hours. RNA was extracted, subjected to an RT-PCR, separated on a 2.0% agarose gel, and transcripts visualized by UV imaging. Protein coding isoforms I/II were significantly upregulated (n=3).
